# Complete Genome Sequence of the Extreme Thermophile *Dictyoglomus thermophilum* H-6-12

**DOI:** 10.1128/genomeA.00109-14

**Published:** 2014-02-20

**Authors:** David A. Coil, Jonathan H. Badger, Heather C. Forberger, Florenta Riggs, Ramana Madupu, Nadia Fedorova, Naomi Ward, Frank T. Robb, Jonathan A. Eisen

**Affiliations:** a University of California Davis Genome Center, Davis, California, USA; b J. Craig Venter Institute, La Jolla, California, USA; c AstraZeneca Pharmaceuticals, Wilmington, Delaware, USA; d Silver Spring, Maryland, USA; e J. Craig Venter Institute, Rockville, Maryland, USA; f University of Wyoming, Laramie, Wyoming, USA; g University of Maryland School of Medicine, Baltimore, Maryland, USA; h University of California Davis, Department of Evolution and Ecology, Department of Medical Microbiology and Immunology, Davis, California, USA

## Abstract

Here, we present the complete genome of the extreme thermophile, *Dictyoglomus thermophilum* H-6-12 (phylum *Dictyoglomi*), which consists of 1,959,987 bp.

## GENOME ANNOUNCEMENT

*Dictyoglomus thermophilum* is an extremely thermophilic, chemo-organotrophic, cellulolytic, obligate anaerobe originally isolated from a hot spring in Japan (1). The cells are rod-shaped, non-spore-forming, nonmotile, and form unusual spherical bodies of up to 100 cells. *D. thermophilum* and *Dictyoglomus turgidum* comprise the only two species in the *Dictyoglomi* phylum. The genome of *D. turgidum* has been sequenced, and the strain is unable to utilize cellulose (2). *D. thermophilum* was selected in 2002 as part of a National Science Foundation-funded “Assembling the Tree of Life” project at The Institute for Genomic Research (TIGR) to sequence the genomes of representatives of the seven phyla of bacteria that at the time had cultured representatives but no available genome sequence.

*D. thermophilum* was obtained from the ATCC, grown, and its DNA was extracted using standard techniques. Sanger sequencing and genome assembly were performed as previously described for genomes sequenced by TIGR (3–5). Small- and large-insert plasmid libraries were constructed in pUC-derived vectors after random mechanical shearing (nebulization) of genomic DNA. Sequencing resulted in 23,127 reads, with an average read length of 790 base pairs. The sequences were assembled using the Celera Assembler (6). The coverage criteria were that every position required at least double-clone coverage (or sequence from a PCR product amplified from genomic DNA) and either sequence from both strands or two different sequencing chemistries. The sequence was edited manually, and additional PCR and sequencing reactions were done to close gaps, improve coverage, and resolve sequence ambiguities (7). All repeated DNA regions were verified by PCR amplification across the repeat and sequencing of the product. The final assembly contains 1,959,987 bp, a G+C content of 34%, and an estimated coverage of ~9×.

The replication origin was determined by the colocalization of genes (*dnaA*, *dnaN*, *recF*, and *gyrA*) often found near the origin in prokaryotic genomes and GC nucleotide skew (G·C/G+C) analysis (8). Completeness of the genome was assessed using the PhyloSift software (9) to sequence for 40 highly conserved single-copy marker genes (10). Thirty-nine of these 40 markers were found in this assembly, and the missing marker (porphobilinogen deaminase) was found in only 80% of the original 1,000 genomes used to generate the markers.

An initial set of open reading frames likely to encode proteins (coding sequences [CDSs]) were predicted as previously described (7). All predicted proteins >30 amino acids were searched against a nonredundant protein database, as previously described (7). Protein membrane-spanning domains were identified by TopPred (11). The 5′ regions of each CDS were inspected to define the initiation codons using similarity searches, as well as the positions of ribosomal binding sites and transcriptional terminators. Two sets of hidden Markov models, Pfam version 11.0 (12) and TIGRFAMs 3.0 (13), were used to determine CDS membership in families and superfamilies. Pfam version 11.0 hidden Markov models were also used, with a constraint of a minimum of two hits to find repeated domains within proteins and mask them.

This resulted in 1,912 predicted protein coding sequences for *D. thermophilum* H-6-12 at the time of submission to Genbank (2008).

### Nucleotide sequence accession numbers.

The genome sequence has been deposited at GenBank under the accession no. CP001146. The version described in this paper is version CP001146.1.
